# Supplementary material for: Selectively biased tri-terminal vertically-integrated memristor configuration
Source: Sci Rep. 2022 Jun 21;12:10467. doi: 10.1038/s41598-022-14462-w (PMC9213395; doi:10.1038/s41598-022-14462-w)
Supplement: Supplementary file 1 — Supplementary Information. [file 41598_2022_14462_MOESM1_ESM.pdf]

Selectively biased tri-terminal vertically-integrated memristor configuration - supplementary

Vasileios Manouras, Spyros Stathopoulos, Alex Serb and Themis Prodromakis

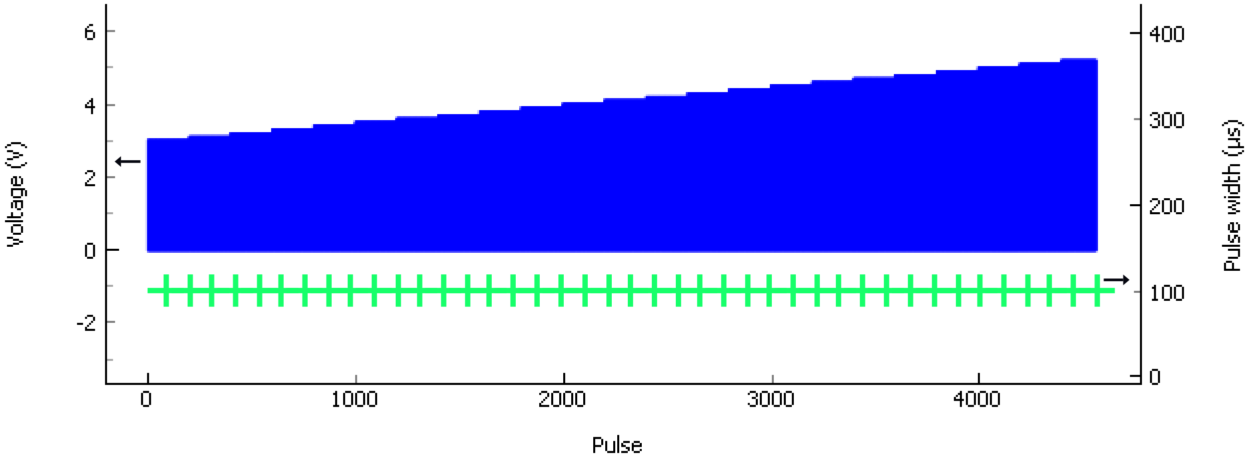

Figure S 1: Pulsing protocol used for electorforming. Blue line denotes a voltage pulse train of increasing amplitude. Green crosses are the pulse width which was always kept steady for electroforming. Voltage amplitude was initiated at 3V and maximum amplitude was 8V. Pulse width was 100µs and amplitude changed every 200 pulses.

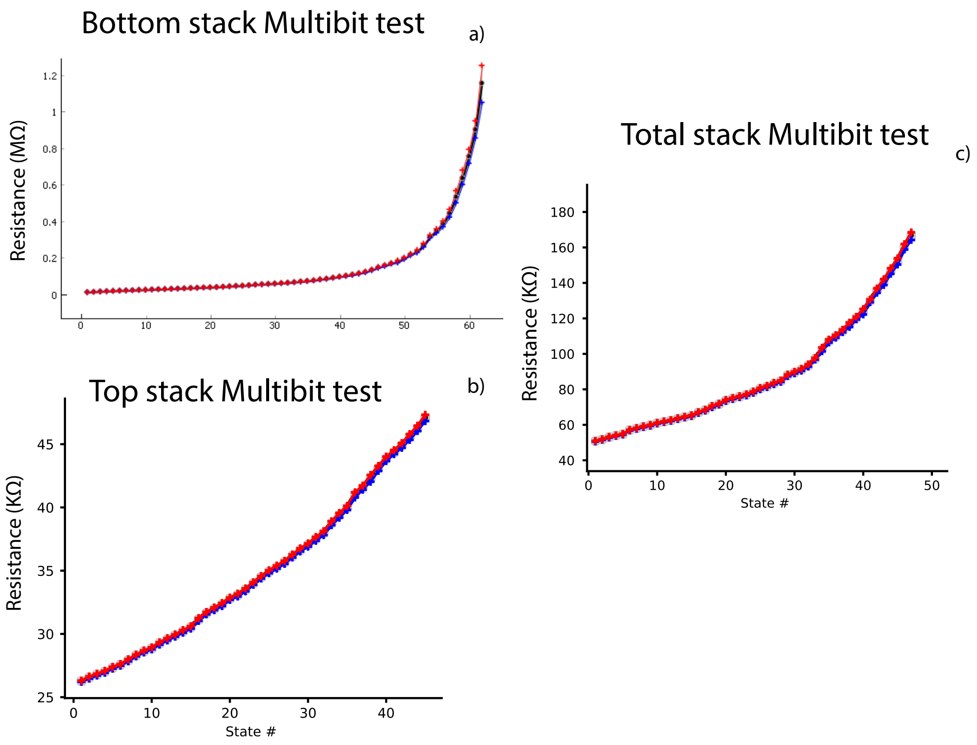

Figure S 2: Multibit protocol pushes the devices with pulses of increasing amplitude or width and registers changes in resistive state. Subfigures (a) and (b) depict the raw output of the protocol, for the individual devices of the total stack which was used in constructing Figure 3 of the main text. A multibit test was also applied to the entire total stack (subfigure c), to ascertain the multistate potential of full stacks. It is apparent that due to the functionality of this protocol, the resistive changes sustained in the individual devices generate a feedback loop, where a device that switches to a higher resistive state will then be more likely to switch again, due to it receiving a bigger share of the input voltage in the subsequent pulse train. Thus it is not possible , in this way, to obtain the full spectrum of resistive states which can be generated by individual switching of each device.

30X30μm^2 Device area

Device	Top Form Pol	Bot Form Pol	Top Switch Pol	Bot Switch Pol
N19	+	−	− ↑	+ ↑
P17	+	−	+ ↑	+ ↑
U12	−	+	+ ↑	− ↑

40X40μm^2 Device area

Device	Top Form Pol	Bot Form Pol	Top Switch Pol	Bot Switch Pol
H25	+	−	+ ↑	+ ↑
J23	−	−	− ↑	+ ↑
T20	−	−	+ ↑	+ ↑
X24	+	−	− ↑	+ ↑
Z26	+	−	+ ↑	+ ↑

50X50μm^2 Device area

Device	Top Form Pol	Bot Form Pol	Top Switch Pol	Bot Switch Pol
H25	+	−	− ↑	+ ↑
T20	−	−	+ ↑	+ ↑

60X60μm^2 Device area

Device	Top Form Pol	Bot Form Pol	Top Switch Pol	Bot Switch Pol
H25	+	−	+ ↑	+ ↑
J23	+	−	− ↑	+ ↑
U12	−	+	− ↑	− ↑
X24	−	−	+ ↑	+ ↑

Figure S 3: This table shows a connection between forming protocol polarity and switching polarity of devices, for the Pt(BE)/[TiO_x/Al_2O_3](AL1)/Pt(ME)/TiO_x(AL2)/Pt(TE) stack. Bottom devices have a [TiO_x/Al_2O_3] active layer, while top devices have a TiO_x active layer. Different device areas where checked. Overall the evidence is overwhelmingly in favour of a connection between forming polarity and switching polarity for bottom devices, with the capping Al2O3 layer. By using a positive polarity to form the devices, a negative switching polarity is developed, and vice versa. On the other hand, this does not seem to be true for top layer devices which only have TiO_x as an active layer. Only 57% of TiO_x devices showed the above-mentioned behaviour, while in the remaining 43% forming of a specific polarity would lead to the device developing the same switching polarity. Thus it seems that forming polarity is irrelevant for this specific material.

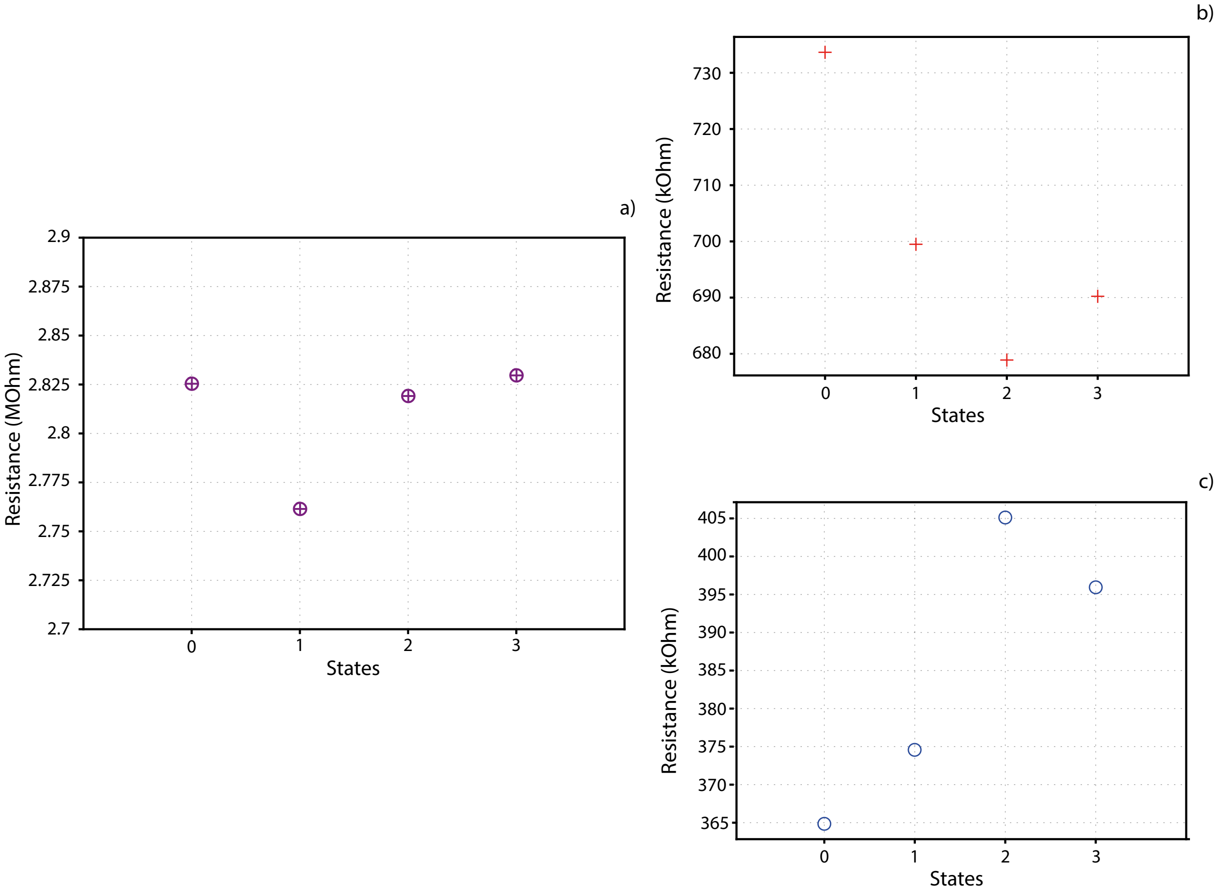

Figure S 4: This supplementary figure represents the second case of anti-serial switching, mentioned in the main text. In contrast with Figure 5 of the main text, application of voltage pulses in the entire stack triggers a response in both devices, which have comparable resistive states and can be switched by the divided stimulus they receive. As a result, the total stack is almost unchanged, with a maximum resistive deviation of 1.7%, as seen in subfigure (a). At the same time, both the Top and Bottom switch inversely, with one increasing and one decreasing when pulsing is applied to them. For state 1 the voltage train pulse had an amplitude of +2.2V, while for state 2 it had +2.3V. This is obvious as both devices keep moving in the same direction as in state 1. State 3 on the other hand sees the application of -2.3V, which does not seem to over-influence the resistive state of the total stack, but in reality both top and bottom devices have reversed part of their previous switching, thus leading to a self balancing system. The top device experiences a resistive change of the order of 7% while the bottom experiences a change up to 9.8%. Both these changes are much higher than the change evident in the full stack. We believe this type of behaviour might be applicable to other devices with multiple layers, such as CRS and multilayered memristors.
